# Oxygen Vacancy and Valence Band Structure of Ba_0.5_Sr_0.5_Fe_1−x_Cu_x_O_3−δ_ (x = 0–0.15) with Enhanced ORR Activity for IT-SOFCs

**DOI:** 10.3390/ma16083231

**Published:** 2023-04-19

**Authors:** Taeheun Lim, Kanghee Jo, Heesoo Lee

**Affiliations:** School of Materials Science and Engineering, Pusan National University, Busan 46241, Republic of Korea; taeheunlim@pusan.ac.kr (T.L.); jokanghee@pusan.ac.kr (K.J.)

**Keywords:** Ba_0.5_Sr_0.5_Fe_1−x_Cu_x_O_3−δ_, oxygen vacancy, valence band, oxygen reduction reaction, area-specific resistance

## Abstract

The oxygen reduction reaction (ORR) activity of a Cu-doped Ba_0.5_Sr_0.5_FeO_3−δ_ (Ba_0.5_Sr_0.5_Fe_1−x_Cu_x_O_3−δ_, BSFCux, x = 0, 0.05, 0.10, 0.15) perovskite cathode was investigated in terms of oxygen vacancy formation and valence band structure. The BSFCux (x = 0, 0.05, 0.10, 0.15) crystallized in a cubic perovskite structure ($Pm\bar{3}m$). By thermogravimetric analysis and surface chemical analysis, it was confirmed that the concentration of oxygen vacancies in the lattice increased with Cu doping. The average oxidation state of B-site ions decreased from 3.583 (x = 0) to 3.210 (x = 0.15), and the valence band maximum shifted from −0.133 eV (x = 0) to −0.222 eV (x = 0.15). The electrical conductivity of BSFCux increased with temperature because of the thermally activated small polaron hopping mechanism showing a maximum value of 64.12 S cm^−1^ (x = 0.15) at 500 °C. The ASR value as an indicator of ORR activity decreased by 72.6% from 0.135 Ω cm^2^ (x = 0) to 0.037 Ω cm^2^ (x = 0.15) at 700 °C. The Cu doping increased oxygen vacancy concentration and electron concentration in the valence band to promote electron exchange with adsorbed oxygen, thereby improving ORR activity.

## 1. Introduction

One of the important challenges for the commercialization of solid oxide fuel cells (SOFCs) is to lower the operating temperature to an intermediate temperature (IT) range of 500–800 °C [1]. The intermediate operating temperature increases the polarization resistance of conventional cathodes like La_1−x_Sr_x_MnO_3_, resulting in reduced electrochemical activity of the cathode for the oxygen reduction reaction (ORR). To solve this problem, cobalt-based oxides with mixed ionic-electronic conductibility (MIEC) have been studied as potential cathode materials for IT-SOFCs [2,3,4]. However, cobalt-based oxides have disadvantages, such as high thermal expansion coefficients (TECs), because of the flexible redox behavior of cobalt and easy evaporation and reduction of cobalt [5,6]. Therefore, developing cobalt-free cathodes with a good ORR activity for IT-SOFCs is needed.

Among the iron-based cathode materials, the cubic BaFeO_3−δ_ (BFO) materials exhibit good oxygen–electron mixed conduction and excellent chemical and thermal stabilities compared with those of cobalt-based materials [7]. The cubic BFO exhibits high oxygen ion conductivity due to the existence of disordered oxygen vacancies and three-dimensional oxygen diffusion pathways [8]. For stabilization of the BFO cubic phase, the A-site was doped with Sr, La, Ce, etc., and Ba_0.5_Sr_0.5_FeO_3−δ_ (BSF) achieved high electronic conductivity [9,10,11]. In addition, the relatively low oxygen reduction reaction rate of Ba_0.5_Sr_0.5_FeO_3−δ_ can be improved by substituting Fe with other transition metals to promote oxygen inflow and oxygen ion transport [12].

Replacing cations with lower valences (2+/3+) for Fe^3+/4+^ cations can form more oxygen vacancies, which can enhance the ORR activity. For this purpose, some researchers have doped Ni, Gd, etc., at the Fe site, and perovskite cubic materials substituted with B-sites such as BaFe_0.75_Ni_0.25_O_3−δ_ and BaF_1−x_Gd_x_O_3−δ_ provided high ORR activity [13,14]. Yin et al. and Lu et al. improved the electrochemical property of Pr_0.5_Sr_0.5_Fe_0.8_Cu_0.2_O_3−δ_ and Nd_0.5_Sr_0.5_Fe_0.8_Cu_0.2_O_3−δ_ by doping Cu into the Fe site [15,16]. In addition, studies on doping Cu with Ni sites have been conducted to improve electrochemical properties. Niemczyk et al. synthesized LaNi_1−x_Cu_x_O_3−δ_ series, and LaNi_0.5_Cu_0.5_O_3−δ_ showed a low polarization resistance of 0.056 Ω cm^2^ at 800 °C and a relatively high power density output of 870 mW cm^−2^ at 900 °C [17]. Jakub et al. studied La_1−x_Sr_x_Ni_1−y_Cu_y_O_3−δ_ and achieved the maximum power value of 450 mW cm^−2^ at 650 °C [18]. Considering that Cu is present in multiple oxidation states (Cu^+^, Cu^2+^, Cu^3+^), similar to Co, Fe, and Mn, it is reasonable to use perovskite materials containing Cu as the SOFC cathode [19].

The mechanism of ORR at the SOFC cathode surface involves four steps, including diffusion and adsorption of oxygen molecules, dissociation of the oxygen molecules to form oxygen atoms, lattice mixing of oxygen ions, and movement of the oxygen ions to the electrode/electrolyte interface [20]. However, the rate-determining step is not well understood because of the complex characteristics of the cathode materials, such as electronic structure and defect structure. Previous studies have suggested descriptors that can predict the ORR activity. Hardin et al. explained that the total number of valence electrons is a descriptor for ORR activity, and Jung et al. predicted the ORR activity by comparing d-band centers with the surface composition and morphology of PtCux@Pt/C catalysts [21,22]. Additionally, Zhu et al. investigated the relationship between the polarization resistance and the valence band of LSCO thin film consisting of Co 3d and O 2p bands [23].

In this study, Ba_0.5_Sr_0.5_Fe_1−x_Cu_x_O_3−δ_ perovskite cathode materials were investigated to confirm the relationship between the change in the valence band structure according to Cu doping and ORR activity. The change in oxygen vacancy concentration of BSFCux according to Cu doping was confirmed through thermogravimetric analysis (TGA) and X-ray photoelectron analysis (XPS). In addition, the oxidation state and the valence band structure were analyzed using XPS and the polarization resistance of BSFCux was calculated as an indication for change in ORR activity via the impedance spectrum.

## 2. Experimental Procedures

### 2.1. Synthesis

Ba_0.5_Sr_0.5_Fe_1−x_Cu_x_O_3−δ_ (BSFCux, x = 0, 0.05, 0.10, 0.15) powders were synthesized by a solid-state reaction using BaCO_3_ (99.0% purity, Sigma-Aldrich, St. Louis, MO, USA), SrCO_3_ (99.9% purity, Sigma-Aldrich), Fe_2_O_3_ (99.0% purity, Sigma-Aldrich), and CuO (99.0% purity, Sigma-Aldrich) as starting materials. After mixing each material according to the stoichiometric ratio, it was ball milled together in a polyethylene container with ethanol and zirconia balls for 24 h and dried at 100 °C for 12 h. The green bodies were prepared using dried powder by uniaxial compression under hydraulic pressure of 20 MPa; the green body of BSF was calcined at 1100 °C for 10 h, and the green bodies of BSFCux (x = 0.05, 0.10, 0.15) were calcined at 950 °C for 10 h. These calcined green bodies were ground using a mortar and sieved using a mesh of 250 μm. Finally, BSFCux powder was synthesized by repeating the calcining, grounding, and sieving process twice.

### 2.2. Characterization

The crystal structure of the synthesized powders was analyzed using powder X-ray diffraction (PXRD, X’pert PRO-MPD, λ = 1.54 Å) in the 2θ range of 20–80° with a step scan procedure (0.02°/2θ step, 1° min^−1^) at room temperature. The structure parameters were obtained by analyzing XRD data with Rietveld refinement (PANalytical X’Pert HighScore Plus software, version 3.0c(3.0.3)). The line shapes of the diffraction peaks were generated by a pseudo-Voigt function and the background refined to a 4th degree polynomial.

In order to determine the temperature of oxygen vacancy formation and weight reduction of BSFCux, thermogravimetric analysis (TGA) was carried out on a thermal analyzer (NETSCH STA 409 PC/PG) with about 100 mg of BSFCux powders measured in a nitrogen atmosphere at a heating rate of 5 °C min^−1^. According to the TGA data, the changes in oxygen non-stoichiometry (δ) for the powder samples were calculated using the following equation [24]:

(1) $$\delta =\delta_{0}+\frac{M_{0}}{15.99}\left(1-\frac{m}{m_{0}}\right)$$

where δ_0_ is the oxygen non-stoichiometry at room temperature, *M*_0_ is the molar mass of the samples with non-stoichiometry δ_0_, 15.99 is the atomic weight of oxygen atoms, *m* is the final weight of the samples under various temperatures, and *m*_0_ is the initial weight of the samples. The δ_0_ was calculated by electroneutrality condition with X-ray photoelectron spectroscopy (XPS, Thermo Fisher Scientific, Waltham, MA, USA) data [25,26,27,28]. XPS was used to analyze changes in oxidation state, oxygen non-stoichiometry, and valence band structure. The spectra were calibrated using the C1s line (BE = 284.6 eV).

The electrical conductivity of BSFCux was evaluated using the 4-probe DC technique in the range of 300–900 °C, and Pt wires were wrapped around the sintered bars with dimensions of 5 × 3 × 30 mm^3^. A direct current of 50 mA was supplied from a current source (Keithley 2400, Solon, OH, USA), and the corresponding voltage drop was collected using a multimeter (Agilent, 34401A, Santa Clara, CA, USA).

The microstructure of the BSFCu0.15|SDC|BSFCu0.15 symmetric cell was observed using a scanning electron microscope (SEM, MIRA3, TESCAN). The area-specific resistance (ASR) of symmetrical cells of BSFCux electrodes was measured by AC impedance spectroscopy. For an impedance analysis, a dense disk of SDC (20 mol% Samarium doped ceria, Fuelcell materials, 20 mm diameter and 600 μm thick) was used as an electrolyte. BSFCux powder was mixed with a vehicle (Fuel Cell Materials) to prepare BSFCux pastes using a three-roll mill, and these pastes were screen-printed on both sides of the SDC pellets with an area of 0.2826 cm^2^. After drying, the symmetric cells were calcined at 900 °C for 2 h in air to improve the adhesion between the electrolyte and the electrode. Electrochemical impedance spectra (EIS) were obtained using Iviumstat (Ivium, Netherlands) equipment, and a 10 mV excitation voltage was applied in the frequency range of 10^6^–10^−2^ Hz at 700 °C. The obtained EIS data were fitted using Zview software version 3.0.

## 3. Results and Discussion

**Figure 1:**
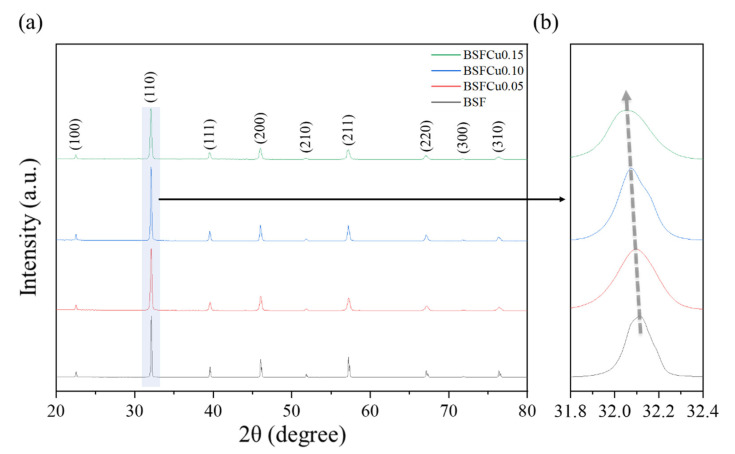
(**a**) Room temperature PXRD patterns of the Ba_0.5_Sr_0.5_Fe_1−x_Cu_x_O_3−δ_ (x = 0, 0.05, 0.10, 0.15). (**b**) Enlarged (110) peaks.

**Figure 2:**
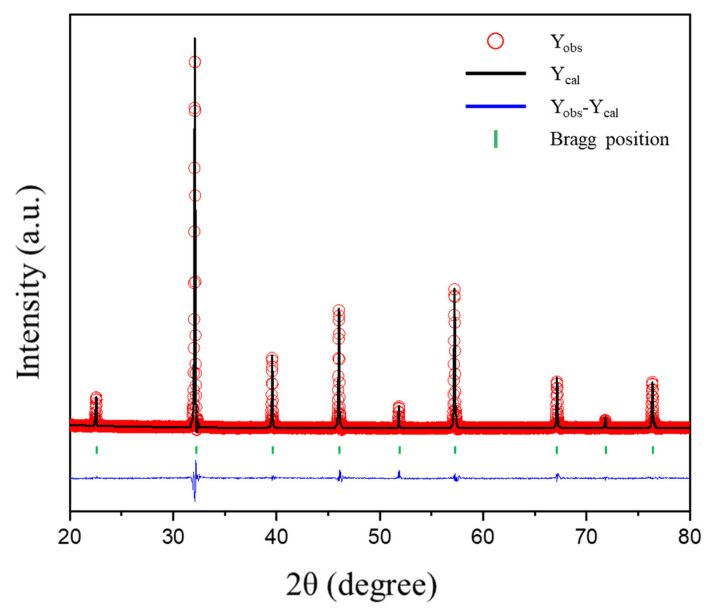
Rietveld refinement result of the Ba_0.5_Sr_0.5_Fe_0.85_Cu_0.15_O_3−δ_ after calcination at 950 °C for 10 h in air. The open symbol is observed intensities, the black line is calculated intensities, the blue line is the difference between the observed and the calculated intensities, and green vertical bars are Bragg positions.

Figure 1a shows the room temperature XRD patterns of the BSFCux (x = 0, 0.05, 0.10, 0.15) powder, and no secondary phases appeared in the patterns, which confirmed that 0–15 mol% of Cu was stably dissolved in the BSF lattice. As shown in Figure 1b, the 2θ value of (110) peak shifted to a smaller angle as the Cu doping increased, which indicated an expansion of the lattice volume due to the larger ionic radii of Cu^+^ (0.77 Å) and Cu^2+^ (0.73 Å) than those of Fe^3+^ (0.645 Å) and Fe^4+^ (0.585 Å). The XRD patterns show that BSFCux crystallizes into a cubic perovskite structure with a space group $Pm\bar{3}m$ (Figure 2). The structural parameters calculated by Rietveld refinement are listed in Table 1, and the lattice volume increased from 61.0699 Å^3^ (x = 0) to 61.4428 Å^3^ (x = 0.15) with the Cu content.

**Figure 3:**
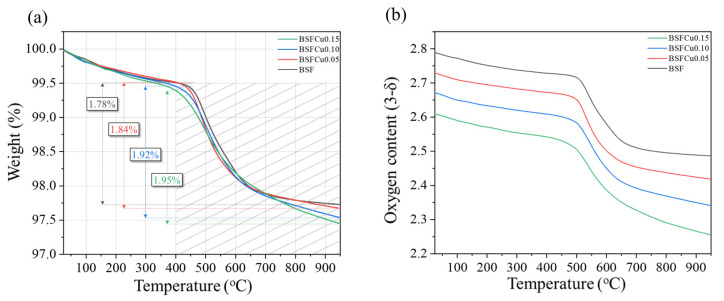
(**a**) Weight change and (**b**) oxygen content curves of the Ba_0.5_Sr_0.5_Fe_1−x_Cu_x_O_3−δ_ (x = 0, 0.05, 0.10, 0.15).

Thermogravimetric analysis was carried out to confirm the effect of Cu doping on oxygen vacancy formation. Figure 3a shows the TG curve of the BSFCux samples from 25 to 950 °C and Figure 3b shows the oxygen content of the BSFCux samples using room-temperature oxygen non-stoichiometry (δ_0_) from the XPS analysis. In order to more clearly observe the oxygen vacancy formation according to the Cu content, it was measured in an inert atmosphere using nitrogen gas. There were weight losses of less than 1% during heating before the slope changing point, which is due to the desorption of physically and chemically adsorbed water and other gases [29]. The slope changing temperature decreased from 500 °C (x = 0) to 450 °C (x = 0.15) and the lower slope changing temperature indicated the easier loss of oxygen in the BSFCu0.15 lattice with increasing temperature. The weight loss after the slope changing temperature increased from 1.72% (x = 0) to 2.09% (x = 0.15). The weight loss observed within this temperature range is due to the loss of lattice oxygen, indicating that the amount of oxygen vacancy in the lattice increases with Cu doping [28].

**Figure 4:**
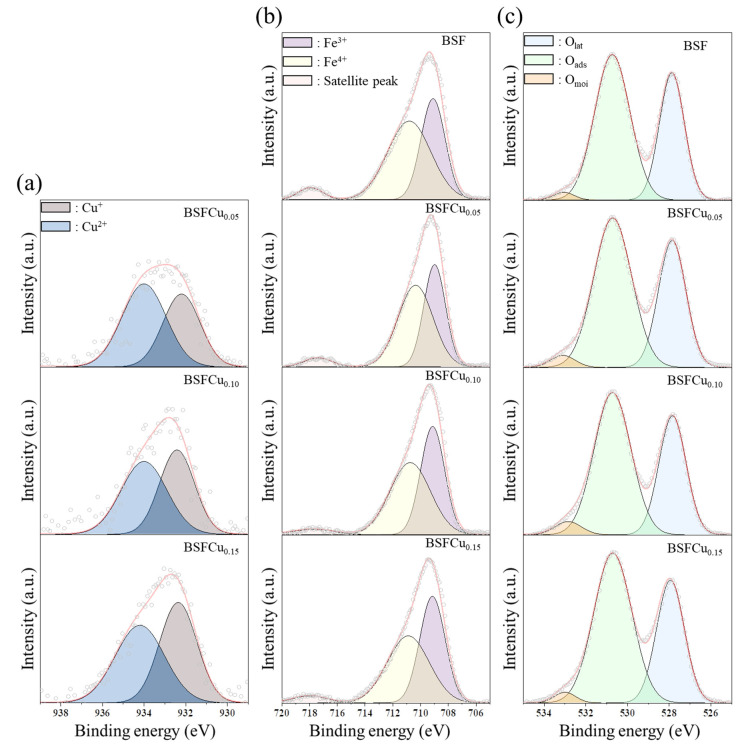
(**a**) Cu 2p_3/2_, (**b**) Fe 2p_3/2_, and (**c**) O 1s XPS spectra of the Ba_0.5_Sr_0.5_Fe_1−x_Cu_x_O_3−δ_ (x = 0, 0.05, 0.10, 0.15). The circles are the experimental data, the red line is the fitting curve.

The X-ray photoelectron spectroscopy (XPS) analysis was performed to characterize the surface chemical bonding state of the BSFCux samples. Figure 4a–c show the Cu 2p_3/2_, Fe 2p_3/2_, and O 1s XPS spectra for each BSFCux composition and the area ratios of peaks are summarized in Table 2. The Cu 2p_3/2_ peak was deconvoluted into two peaks at approximately 932.3 eV (Cu^+^) and 934.1 eV (Cu^2+^), as shown in Figure 4a [30]. The Fe 2p_3/2_ peak was deconvoluted into 709.1 eV (Fe^3+^) and 710.8 eV (Fe^4+^) peaks, and a weak satellite shake-up peak was observed in the range of 715 eV to 720 eV, as shown in Figure 4b [31]. The average oxidation states of the B-site ions (Fe and Cu) are 3.583 (x = 0), 3.464 (x = 0.05), 3.332 (x = 0.10), and 3.210 (x = 0.15). Assuming an oxidation state of 2^+^ for Ba and Sr, the room-temperature oxygen non-stoichiometry (δ_0_) was calculated according to the electroneutrality condition, and the oxygen non-stoichiometry increased from 0.21 (x = 0) to 0.39 (x = 0.15) as the Cu doping increased (Table 2). It is thought that the BSFCu0.15 cathode will enhance the catalytic activity of the ORR because the value of δ_0_ indicates the amount of oxygen vacancies in the lattice.

In Figure 4c, the O 1s peak was deconvolved into three peaks of 527.9 eV, 530.7 eV, and 533 eV corresponding to lattice oxygen (O_lat_), adsorbed oxygen (O_ads_), and surface moisture (O_moi_), respectively [32]. The adsorbed oxygen is released easily from the surface of the crystal lattice with increasing temperature, which leads to the oxygen vacancy formation. Therefore, the area ratio of O_asd_ to O_lat_ (O_ads_/O_lat_) can be used as a criterion to evaluate the relative content of oxygen vacancies in materials [33,34]. The O_ads_/O_lat_ were 1.54 (x = 0), 1.60 (x = 0.05), 1.62 (x = 0.10), and 1.65 (x = 0.15), which increased with the amount of Cu doping. It can be interpreted that BSFCu0.15 has the highest oxygen vacancy concentration among the BSFCux (x = 0, 0.05, 0.10, 0.15) in the intermediate temperature region, which corresponds with the thermogravimetric results (Figure 3a,b).

**Figure 5:**
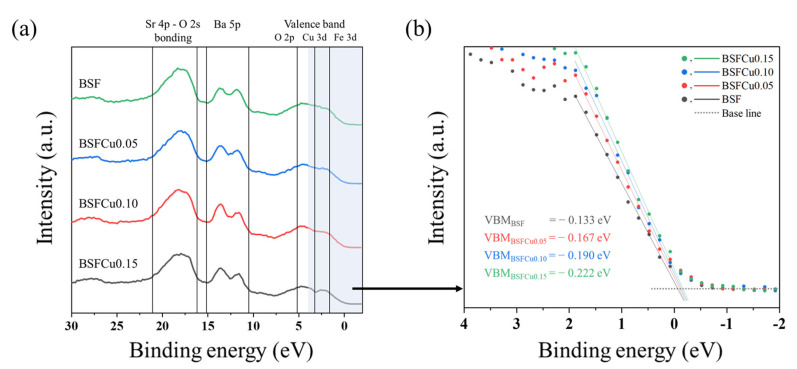
(**a**) Valence band (VB) spectra normalized to the integrated intensity for BSFCux; (**b**) linear fit of the leading edge of VB used to obtain the valence band maximum (VBM).

The valence band (VB) spectra of BSFCux are shown in Figure 5, and the peak of the Ba 5p (10.5–15.0 eV) and Sr 4p-O 2s bond (16–21 eV) was confirmed [35]. The range of 0–7.5 eV is made up of Fe 3d (1.8 eV), Cu 3d (3.1 eV), and O 2p orbitals (5.0 eV), which is closely related to the BO6 structure. The Cu 3d area increased with an increase in Cu doping, leading to a negative shift in the valence band maximum (VBM) from −0.133 eV (BSF) to −0.222 eV (BSFCu0.15), as shown in Figure 5b. The negative shift of the VBM value represents that electron transport to the adsorbed oxygen is improved because of the increased electron concentration in the VB of BSFCux, which is expected to enable faster oxygen exchange.

**Figure 6:**
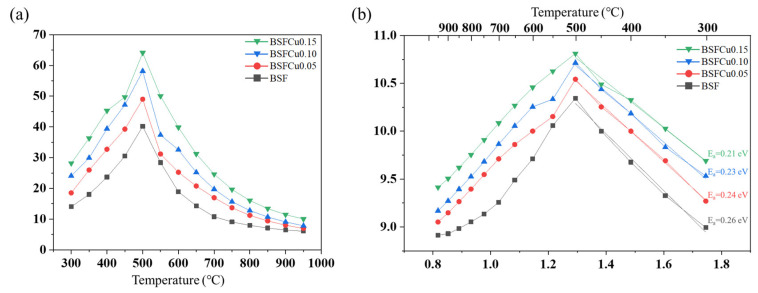
(**a**) The temperature dependence of electrical conductivity of the Ba_0.5_Sr_0.5_Fe_1−x_Cu_x_O_3−δ_ (x = 0, 0.05, 0.10, 0.15) measured from 300 °C to 950 °C in air; (**b**) ln(σT) vs. 1000/T plot.

Fe-based perovskite oxides show mixed ionic-electronic conductivity (MIEC) because of the simultaneous presence of oxygen vacancies and electron holes as charge carriers. However, since the electrical conductivity is about two orders of magnitude higher than the ionic conductivity, the conductivity data of Figure 6 mainly represent the electronic conductivity [15,36]. The electrical conductivity (σ) of BSFCux was measured in air at 300–950 °C using the 4-probe DC technique (Figure 6a). The electrical conductivity of BSFCux increased with temperature and reached a maximum at 500 °C with 64.12 S cm^−1^ (x = 0.15). This semiconducting behavior can be explained by a thermally activated small polaron hopping mechanism [37]. The decrease in electrical conductivity is related to the breakdown of (Fe, Cu)–O–(Fe, Cu) bonds above 500 °C because the oxygen vacancies are formed and the charge carrier concentration is reduced due to the reduction in Fe^4+^ (Equation (2)), which is in good agreement with the thermogravimetric results [38].

(2) $$2Fe_{Fe}^{•}+O_{O}^{\times }↔2Fe_{Fe}^{\times }+V_{O}^{••}+\frac{1}{2}O_{2}↑$$

where $Fe_{Fe}^{•}$ and $Fe_{Fe}^{\times }$ represent the Fe^4+^ and Fe^3+^, $O_{O}^{\times }$ and $V_{O}^{••}$ represent the lattice oxygen and oxygen vacancy.

Figure 6b shows the Arrhenius plot for the electrical conductivity of BSFCux, and the relationship between electrical conductivity (*σ*) and temperature follows the Arrhenius equation [39]:

(3) $$\sigma =A\exp\left(-\frac{E_{a}}{kT}\right)$$

where *A*, *E_a_*, *k*, and *T* are the pre-exponential constant, activation energy, Boltzman constant, and temperature, respectively. According to the slope of linear fit over the temperature of 300–500 °C, the activation energy (*Ea*) was 0.26 eV (x = 0), 0.24 eV (x = 0.05), 0.23 eV (x = 0.10), and 0.21 eV (x = 0.15).

The low activation energy value helps to improve the hopping of the charge carrier, thereby increasing the electrical conductivity.

**Figure 7:**
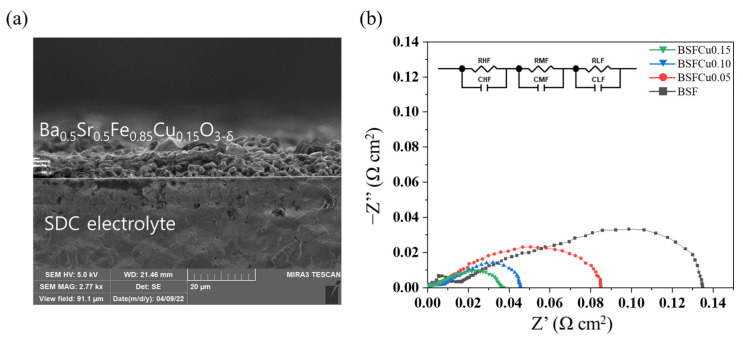
(**a**) Cross-section view of SEM image of the BSFCu0.15|SDC|BSFCu0.15 symmetric cell; (**b**) impedance spectra of the BSFCux|SDC|BSFCux symmetric cell measured at 700 °C in air.

The cross-section view of the BSFCu0.15|SDC|BSFCu0.15 symmetrical cell shows that the interface contact is tightly formed between the porous electrode and the dense electrolyte (Figure 7a). The ORR catalytic activity of BSFCux cathodes was assessed via electrochemical impedance spectroscopy of the symmetric cell configuration (BSFCux|SDC|BSFCux), and the EIS plots measured at 700 °C in air are shown in Figure 7b, where an ohmic resistance was subtracted. In order to clarify the ORR mechanism, the impedance spectra were fitted with the equivalent circuit model (R_HF_//CPE_HF_)-(R_MF_//CPE_MF_)-(R_LF_//CPE_LF_), which is inserted in Figure 7b. R and CPE in the parallel (R//CPE) represent the polarization resistance and constant phase element, respectively, and the corresponding results are displayed in Table 3.

These impedance spectra consist of high-, medium-, and low-frequency arcs. In general, the high-frequency (HF) arc is related to the process of diffusion of oxygen ions from the triple-phase boundaries into the electrolyte, the medium-frequency (MF) arc is associated with the oxygen surface exchange, and the low-frequency (LF) arc is attributed to the gas diffusion process and oxygen adsorption–dissociation process [40]. In all cases, the low-frequency arc shows the highest polarization resistance, indicating that it was the rate-determining step of ORR. Among the three polarization resistances, R_MF_ decreased the most, by about 81%, from 0.285 Ω (x = 0) to 0.054 Ω (x = 0.15), and it is thought that this is because the oxygen surface exchange reaction was accelerated by increasing the electron concentration of VB with Cu doping.

The area-specific resistance (*ASR*) can be calculated by measuring the distance between the intercepts of Z′ or by using the equation as follows:

(4) $$ASR=R_{p}\frac{active\;area\;of\;cathode}{2}$$

where *R_p_* = R_HF_ + R_MF_ + R_LF_ and the active area of the cathode is 0.2826 cm^2^. The *ASR* of the BSFCux was 0.135 Ω cm^2^ (x = 0), 0.085 Ω cm^2^ (x = 0.05), 0.045 Ω cm^2^ (x = 0.10), and 0.037 Ω cm^2^ (x = 0.15). The BSFCu0.15 showed a 72.6% smaller value compared to that of BSF, which can be explained by higher oxygen vacancy concentration and electrical conductivity. A comparison of the ASRs for different iron-based electrodes is listed in Table 4. It can be concluded that the BSFCu0.15 cathode has high electrocatalytic activity for ORR due to improved interaction with adsorbed oxygen with an increase in oxygen vacancies at intermediate temperature.

## 4. Conclusions

The oxygen reduction reaction (ORR) activity of a Ba_0.5_Sr_0.5_Fe_1−x_Cu_x_O_3−δ_ (BSFCux, x = 0, 0.05, 0.10, 0.15) perovskite cathode was investigated. BSFCux was crystallized into a cubic perovskite structure with a $Pm\bar{3}m$ space group, and it was confirmed that the lattice volume of the BSFCux increased with Cu doping. For thermogravimetric and XPS analysis, the concentration of oxygen vacancies in the lattice increased with increasing Cu doping. The valence band maximum shifted from −0.133 eV (x = 0) to −0.222 eV (x = 0.15), which promoted the electron transfer from the cathode surface to adsorbed oxygen molecules. The electrical conductivity of BSFCux increased with temperature by the thermally activated small polaron hopping mechanism, showing a maximum value of 64.12 S cm^−1^ (x = 0.15) at 500 °C. The ASR of BSFCu0.15 was 72.6% smaller compared to that of BSF, which can be explained by higher oxygen vacancy concentration and electrical conductivity. The ORR activity of BSFCux increased with Cu doping due to an increase in oxygen vacancy concentration and electron concentration in the valence band, which promotes electron exchange between the cathode surface and adsorbed oxygen.

## Figures and Tables

**Table 1:** Structural parameters of the Ba_0.5_Sr_0.5_Fe_1−x_Cu_x_O_3−δ_ (x = 0, 0.05, 0.10, 0.15) calculated by Rietveld refinement of the room-temperature XRD data.

Parameters	Composition			
	BSF	BSFCu0.05	BSFCu0.10	BSFCu0.15
a = b = c [Å]	3.938(6)	3.940(4)	3.943(7)	3.946(5)
Volume [Å^3^]	61.0699(9)	61.1630(1)	61.3028(6)	61.4428(3)
Space group	$Pm\bar{3}m$	$Pm\bar{3}m$	$Pm\bar{3}m$	$Pm\bar{3}m$
R_wp_ [%]	6.285	7.211	7.412	6.568
R_exp_ [%]	3.792	3.281	3.414	3.364
χ^2^	2.747	4.832	4.715	3.811

**Table 2:** Fitting results of the Fe 2p_3/2_, Cu 2p_3/2_, and O 1s XPS spectra of the Ba_0.5_Sr_0.5_Fe_1−x_Cu_x_O_3−δ_ (x = 0, 0.05, 0.10, 0.15).

Sample	Fe^3+^ (%)	Fe^4+^ (%)	Cu^+^ (%)	Cu^2+^ (%)	Average Oxidation State	δ_0_	O_lat_ (%)	O_ads_ (%)	O_moi_ (%)	O_ads_/O_lat_
BSF	41.7	58.3	-	-	+3.583	0.21	38.6	59.5	1.9	1.54
BSFCu0.05	43.7	56.3	42.6	57.4	+3.464	0.27	37.3	59.6	3.1	1.60
BSFCu0.10	46.8	53.2	46.9	53.1	+3.332	0.33	36.7	59.5	3.8	1.62
BSFCu0.15	48.9	51.1	49.5	50.5	+3.210	0.39	36.6	60.3	3.1	1.65

**Table 3:** The Rp fitting results of the Ba_0.5_Sr_0.5_Fe_1−x_Cu_x_O_3−δ_ (x = 0, 0.05, 0.10, 0.15) cathode at 700 °C.

Sample	R_HF_ (Ω)	R_MF_ (Ω)	R_LF_ (Ω)	Rp (Ω)	ASR (Ω cm^2^)
BSF	0.141	0.285	0.529	0.955	0.135
BSFCu0.05	0.081	0.170	0.350	0.602	0.085
BSFCu0.10	0.073	0.100	0.145	0.318	0.045
BSFCu0.15	0.034	0.054	0.173	0.262	0.037

**Table 4:** ASRs for different iron-based electrodes.

Cathode	Electrolyte	Operating Temperature (°C)	ASR (Ω cm^2^)	Ref.
La_0.6_Ca_0.4_Fe_0.8_Ni_0.2_O_3−δ_	SDC	750	0.14	[41]
Bi_0.5_Sr_0.5_FeO_3−δ_	SDC	700	0.12	[42]
Sm_0.5_Sr_0.5_Fe_0.8_Cu_0.2_O_3−_*_δ_*	SDC	700	0.084	[43]
Ba_0.5_Sr_0.5_Fe_0.9_Nb_0.1_O_3−_*_δ_*	SDC	700	0.082	[44]
BSFCu0.15	SDC	700	0.037	This work

## Data Availability

Data available upon reasonable request.
